# Body Mass Index Subclassification and Future Risk of Metabolic Dysfunction–Associated Steatotic Liver Disease and Liver-Related Events

**DOI:** 10.1016/j.tjnut.2026.101386

**Published:** 2026-01-29

**Authors:** Shunming Zhang, Daniel E Coral, Zhenyu Huo, Yan Borné, Ming-Hua Zheng, Tao Huang, Lu Qi

**Affiliations:** 1 School of Public Health, Xi’an Jiaotong University Health Science Center, Xi’an, China; 2 Nutritional Epidemiology, Department of Clinical Sciences Malmö, Lund University, Malmö, Sweden; 3 Genetic and Molecular Epidemiology Unit, Lund University Diabetes Centre, Department of Clinical Science Malmö, Lund University, Malmö, Sweden; 4 Department of Gastroenterology, Beijing Friendship Hospital, Capital Medical University, Beijing, China; 5 MAFLD Research Center, Department of Hepatology, the First Affiliated Hospital of Wenzhou Medical University, Wenzhou, China; 6 Key Laboratory of Diagnosis and Treatment for the Development of Chronic Liver Disease in Zhejiang Province, Wenzhou, China; 7 Department of Epidemiology and Biostatistics, School of Public Health, Peking University, Beijing, China; 8 Department of Epidemiology, Celia Scott Weatherhead School of Public Health and Tropical Medicine, Tulane University, New Orleans, LA, United States; 9 Department of Nutrition, Harvard T.H. Chan School of Public Health, Boston, MA, United States

**Keywords:** obesity, metabolic dysfunction–associated steatotic liver disease, liver-related events, BMI, cardiometabolic biomarker

## Abstract

**Background:**

Recent research using a data-driven cluster approach has identified 5 discordant subclassifying body mass index (BMI) subgroups, characterized by cardiometabolic biomarkers deviated from those predicted by BMI.

**Objectives:**

This study aimed to investigate the associations of these subgroups with risks of metabolic dysfunction–associated steatotic liver disease (MASLD) and liver-related events (LREs).

**Methods:**

This prospective cohort study included 423,091 participants. The same cluster analysis as reported by Coral et al. was performed to classify subpopulations. Incident MASLD and LREs were determined by electronic health records. Cox proportional hazards models were used to evaluate the hazard ratio (HR) and 95% confidence interval (CI).

**Results:**

Profiles derived from the study were similar to those identified in the work by Coral et al. Individuals with discordantly high liver transaminase [HR (95% CI): 1.72 (1.51, 1.96) for MASLD and 1.42 (1.23, 1.65) for LREs in males and 1.92 (1.61, 2.28) for MASLD and 1.68 (1.32, 2.14) for LREs in females] and hyperglycemia [HR (95% CI): 1.36 (1.06, 1.74) for MASLD and 1.31 (1.01, 1.70) for LREs in males and 1.62 (1.27, 2.08) for MASLD and 1.80 (1.31, 2.47) for LREs in females] had higher risks of liver outcomes compared with the concordant profile. In contrast, we observed a lower risk of MASLD [HR (95% CI): 0.71 (0.60, 0.84)] in females with discordantly high blood pressure relative to their BMI. For discordant adverse lipid profile and discordant inflammatory profile, no significant associations were observed. In addition, the BMI subclassification profiles had better predictive ability among males.

**Conclusions:**

Metabolically distinct BMI subgroups exhibit heterogeneous risks of MASLD and LREs.

## Introduction

Liver disease accounts for >2 million deaths per year and is responsible for 4% of all deaths worldwide [1]. Metabolic dysfunction–associated steatotic liver disease (MASLD) is currently the most common chronic liver disease globally that affects ∼30% of the world’s adult population [2]. MASLD has emerged as the primary cause of cirrhosis, hepatocellular carcinoma, liver transplantation, and liver-related mortality [3]. Because of the increase in the prevalence of metabolic risk factors and the aging population, the burden of adverse liver-related events (LREs) due to MASLD will continue to grow in the following decades [1]. Therefore, early prevention of MASLD and LREs is a high public health priority.

Obesity is a major risk factor for MASLD and LREs [[4], [5], [6]]. However, not all individuals with obesity progress to liver diseases [7]. Indeed, people with a similar body mass index (BMI, in kg/m^2^), a common obesity measure [8], exhibit varying degrees of liver disease risk. Therefore, distinguishing subpopulations whose metabolic risk diverges from what is expected based on BMI may help facilitate precision prevention strategies for liver diseases. In this regard, a recent study used an ensemble of clustering techniques to identify 5 replicable discordant profiles of individuals whose cardiometabolic biomarkers dissimilarly deviated from those predicted by BMI in 4 large independent cohorts across Europe [9]. The 5 distinct profiles were characterized by differences in blood glucose, serum creatinine (SCR), lipid profile, blood pressure (BP), alanine aminotransferase (ALT), C-reactive protein (CRP), and waist-to-hip ratio (WHR) relative to their BMI. These profiles present a more systematic evaluation of the various obesity-related cardiometabolic phenotypes. Moreover, the 5 discordant profiles or subpopulations exhibit heterogeneous associations with risks of cardiovascular disease (CVD) and type 2 diabetes (T2D) [9]. However, whether similar heterogeneity persists for the relation between these metabolically discordant profiles and liver diseases remains unclear. Given the overlap in the pathophysiologic processes of CVD, T2D, and liver diseases [10,11], we hypothesized that subpopulations with discordant cardiometabolic profiles would experience different risks of liver diseases. Confirming this hypothesis would be conducive to targeted risk factor management for the early and precision prevention of liver diseases.

In the current study, we evaluated how these distinct profiles were associated with risks of incident MASLD and LREs in the UK Biobank.

## Methods

### Study population

The UK Biobank is an ongoing prospective cohort consisting of >500,000 adults aged 37–73 y when enrolled between 2006 and 2010 in England, Scotland, and Wales [12]. Participants were invited to complete touchscreen questionnaires, undergo physical measures, provide biological samples, and receive longitudinal follow-up through linkages to routinely available national health care systems. The research was approved by the North West Multicenter Research Ethics Committee (11/NW/0382), and conducted in accordance with both the Declarations of Helsinki and Istanbul. All the participants signed informed consent. This study excluded participants with missing data on cardiometabolic biomarkers or smoking status, as well as those with prevalent MASLD or LREs at baseline (Supplementary Figure 1). The sample size (total: 423,091; males: 195,168; females: 227,923) was determined based on the availability of data. According to the principle of 10 outcome events per variable [13], this sample size was large enough to provide adequate statistical power. This manuscript followed the STROBE reporting guidelines [14].

### Assessment of cardiometabolic biomarkers

The cardiometabolic biomarkers used in the study included BMI, WHR, systolic BP (SBP), diastolic BP (DBP), blood glucose, SCR, ALT, CRP, high-density lipoprotein (HDL) cholesterol, triglycerides (TG), and low-density lipoprotein (LDL) cholesterol. BMI and WHR (cm/cm) were calculated based on anthropometric measurements obtained during the initial assessment center visit. The averages of 2 BP measures taken a few moments apart were recorded as the final BP values; a manual sphygmometer was used when the standard automated device could not be used. Baseline blood samples were used to measure blood glucose and SCR, as well as serum ALT, CRP, HDL cholesterol, TG, and LDL cholesterol concentrations.

### Ascertainment of MASLD and LREs and follow-up

The study outcomes were incident MASLD and LREs, which were obtained through linked hospital inpatient records and death registers. Incident MASLD was assessed using the International Classification of Diseases, Tenth Revision (ICD-10) codes K76.0 and K75.8 [15]. Incident LREs were a composite endpoint of hepatocellular carcinoma, hepatic decompensation (including ascites, variceal hemorrhage, hepatic encephalopathy, and hepatorenal syndrome), liver transplantation, and liver-related death [16,17]. The diagnostic ICD-10 codes are shown in Supplementary Table 1. The end of follow-up was 30 September, 2021, for participants in England, 28 February, 2018, for Wales, and 31 July, 2021, in Scotland.

### Assessment of covariates

Touchscreen questionnaires were used to collect baseline information on age (continuous: years), sex (categorical: male or female), ethnicity (White or others), Townsend deprivation index (continuous), smoking (categorical: current or noncurrent), and alcohol intake (continuous: g/wk) [18], physical activity [using the International Physical Activity Questionnaire; categorized as low (<600), moderate (600–3000), high (≥3000), or missing based on metabolic equivalent task min/wk for all activity], sedentary time (continuous: h/d), use of antihypertensive agents (categorical: yes or no), use of antidiabetic agents (categorical: yes or no), use of lipid-lowering agents (categorical: yes or no), and personal history of disease [including CVD and cancer (categorical: each yes or no)]. Prevalent CVD (categorical: yes or no) was identified by self-reported disease history and ICD-10 codes (I20-I25 and I60-I64). Dietary data were collected using the touchscreen questionnaire, which asked 29 questions about the average frequency of consumption of main foods and food groups over the past year [19]. A healthy diet score (continuous: 0–5 scores) was derived from 5 food groups (vegetables, fruits, fish, unprocessed red meats, and processed meats), as described in our recent work [20].

### Cluster analysis

To identify discordant profiles consisting of individuals with cardiometabolic biomarkers higher or lower than expected given their BMI, we first repeated the sex-specific cluster analysis published by Coral et al. [9] using the partitioning algorithm. In brief, linear regression models were fitted for each cardiometabolic biomarker (including WHR, SBP, DBP, blood glucose, SCR, ALT, CRP, HDL cholesterol, TG, and LDL cholesterol) where the biomarker was the dependent variable and BMI was the independent variable, adjusting for age and smoking status. As detailed in previous studies [9,21], the model residuals (calculated as the observed biomarker value minus BMI-predicted values) were extracted, standardized, and used to generate the cluster parameters based on the partitioning algorithm. Such an algorithm showed better partition quality than other clustering algorithms, including centroid-based (Gaussian mixture), boundary-based (archetypes), and density-based algorithms [9]. In the subsequent analyses, we selected 6 validated profiles, including 1 concordant and 5 discordant profiles [9]. Each participant was classified into a unique profile exhibiting the maximal profile probability, consistent with a recent study [21].

### Statistical analysis

Baseline characteristics of the study participants were presented as mean ± standard deviation (SD) for continuous variables or as frequency (%) for categorical variables.

The follow-up period started from the date of enrollment until the date of the first occurrence of the outcomes, death, loss to follow-up, or the end of follow-up. The multivariable Cox proportional hazards model was used to assess risk of incident MASLD and LREs associated with the discordant profiles, with the concordant profile as the reference group. Using Schoenfeld residuals, no violation of the proportional hazards assumption was found. The sex-specific models were adjusted for age, ethnicity, Townsend deprivation index, smoking, alcohol intake, healthy diet score, physical activity, sedentary time, use of antihypertensive agents, use of antidiabetic agents, use of lipid-lowering agents, CVD, cancer, and cardiometabolic biomarkers (including BMI, WHR, SBP, DBP, blood glucose, SCR, ALT, CRP, HDL cholesterol, TG, and LDL cholesterol). Missing values of covariates were imputed with medians for continuous variables and represented using indicator variables for categorical variables. The percentage of missing values for covariates is shown in Supplementary Table 2. Furthermore, Kaplan–Meier curves were used to plot the cumulative incidence of each outcome over time in the identified profiles.

The likelihood ratio test was used to compare models with and without profile allocations. The predictive performance of the identified profiles for incident MASLD and LREs was assessed by both discrimination (*c*-statistic) and calibration measures, including the net reclassification improvement index [22] and integrated discrimination improvement. The net reclassification improvement index quantified the incremental prognostic value gained when adding these profiles to the reference model (the above multivariable Cox model without including the identified profiles). The integrated discrimination improvement quantified the difference in predicted probabilities between 2 models. In addition, the decision curve analysis [23] and calibration plot were used to assess the added net benefit of discordant profiles. Moreover, to assess whether these profile allocations survive when selecting the optimal penalty parameters, we performed Lasso Cox regression with 10-fold cross-validation on the models with profile allocations and covariables. Because there are no available thresholds, continuous profile allocations were used in the above-mentioned analyses.

To confirm the robustness of the results, several sensitivity analyses were performed. First, we excluded individuals with MASLD or LREs that occurred within the first 2 y of follow-up to minimize reverse causation. Second, we estimated hazard ratios (HRs) and 95% confidence intervals (CIs) associated with shifting 10% probability from the concordant profile to each of the discordant profiles instead of assigning participants to a specific profile based on the maximal profile probability in the primary analysis. Finally, we used Fine–Gray regression models to estimate the associations, accounting for death as a competing risk.

All statistical analyses were conducted using R version 4.4.2 and SAS version 9.4 (SAS Institute Inc.). Two-sided *P* values <0.05 were considered statistically significant. For multiple testing, the Benjamin–Hochberg method was used to control the false discovery rate at a 5% level.

## Results

### Baseline characteristics of study participants

Table 1 shows the baseline characteristics of study participants overall and by sex. Among the study sample of 423,091 participants, 195,168 (46.1%) were males, and 399,583 (94.4%) were of White ethnicity. The means ± SDs of the study participants were 56.6 ± 8.1 y for age and 27.4 ± 4.8 kg/m^2^ for BMI. Furthermore, the distribution of variables across validated profiles is displayed in Supplementary Table 3 for males and Supplementary Table 4 for females.

**TABLE 1:** Baseline characteristics of the study participants^1^

Characteristics	Overall	Male	Female
Number of participants (n)	423,091	195,168	227,923
Age (y)	56.55 ± 8.09	56.74 ± 8.20	56.38 ± 8.00
White ethnicity [n (%)]	399,583 (94.44)	184,125 (94.34)	215,458 (94.53)
Townsend deprivation index	−1.33 ± 3.07	−1.29 ± 3.13	−1.36 ± 3.03
Body mass index (kg/m^2^)	27.42 ± 4.76	27.83 ± 4.23	27.06 ± 5.15
Waist circumference (cm)	90.31 ± 13.43	96.91 ± 11.30	84.66 ± 12.50
Waist-to-hip ratio	0.87 ± 0.09	0.94 ± 0.07	0.82 ± 0.07
Systolic blood pressure (mmHg)	137.96 ± 18.63	141.00 ± 17.47	135.35 ± 19.20
Diastolic blood pressure (mmHg)	82.31 ± 10.15	84.14 ± 10.02	80.74 ± 9.99
Blood glucose (mmol/L)	5.12 ± 1.23	5.18 ± 1.39	5.06 ± 1.06
Serum creatinine (μmol/L)	72.32 ± 18.05	81.72 ± 18.63	64.27 ± 12.92
Alanine aminotransferase (U/L)	23.50 ± 14.06	27.40 ± 15.10	20.16 ± 12.15
C-reactive protein (mg/L)	2.59 ± 4.32	2.46 ± 4.31	2.70 ± 4.33
High-density lipoprotein cholesterol (mmol/L)	1.45 ± 0.38	1.28 ± 0.31	1.59 ± 0.38
Low-density lipoprotein cholesterol (mmol/L)	3.56 ± 0.87	3.48 ± 0.86	3.63 ± 0.87
Triglycerides (mmol/L)	1.74 ± 1.02	1.97 ± 1.14	1.55 ± 0.86
Current smoking [n (%)]	44,513 (10.52)	24,273 (12.44)	20,240 (8.88)
Alcohol intake (g/wk)	118.52 ± 125.37	158.09 ± 147.33	81.42 ± 85.19
Healthy diet score	3.43 ± 1.17	3.13 ± 1.19	3.67 ± 1.09
Sedentary time (h/d)	4.81 ± 2.44	5.30 ± 2.65	4.38 ± 2.16
Physical activity [n (%)]			
Low	63,748 (15.07)	30,850 (15.81)	32,898 (14.43)
Moderate	173,073 (40.91)	80,876 (41.44)	92,197 (40.45)
High	105,588 (24.96)	53,277 (27.30)	52,311 (22.95)
Missing	80,682 (19.07)	30,165 (15.46)	50,517 (22.16)
Cardiovascular disease [n (%)]	29,553 (6.99)	19,320 (9.90)	10,233 (4.49)
Cancer [n (%)]	36,302 (8.58)	13,110 (6.72)	23,192 (10.18)
Use of antihypertensive agents [n (%)]	87,996 (20.80)	47,865 (24.53)	40,131 (17.61)
Use of antidiabetic agents [n (%)]	4504 (1.06)	2705 (1.39)	1799 (0.79)
Use of lipid-lowering agents [n (%)]	73,647 (17.41)	44,667 (22.89)	28,980 (12.71)

1 Data are expressed as mean ± standard deviation for continuous variables or as frequency (%) for categorical variables.

### The concordant and discordant profiles

**FIGURE 1:**
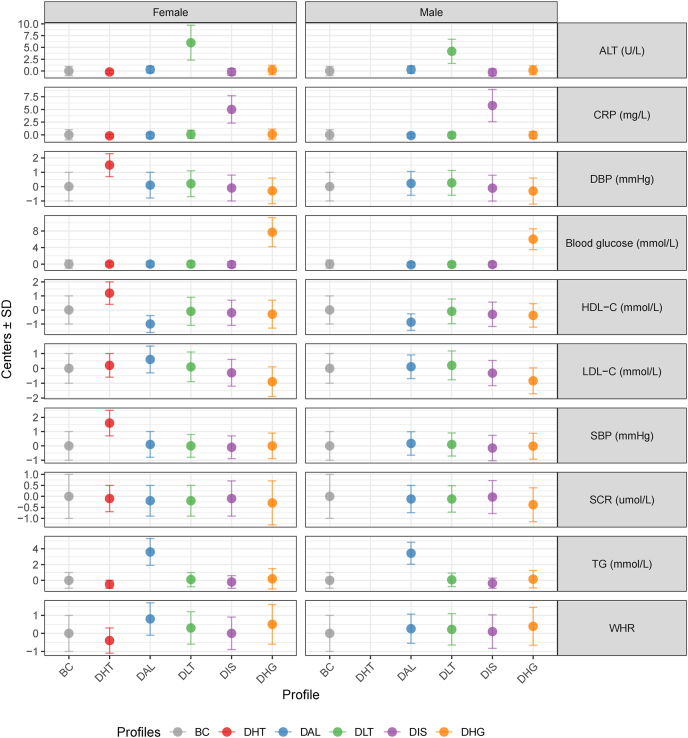
Centers for the identified profiles. ALT, alanine aminotransferase; BC, baseline concordant; CRP, C-reactive protein; DAL, discordant adverse lipid; DBP, diastolic blood pressure; DHG, discordant hyperglycemic; DHT, discordant hypertensive; DIS, discordant inflammatory state; DLT, discordant liver transaminase; HDL-C, high-density lipoprotein cholesterol; LDL-C, low-density lipoprotein cholesterol; SBP, systolic blood pressure; SCR, serum creatinine; TG, triglycerides; WHR, waist-to-hip ratio.

**FIGURE 2:**
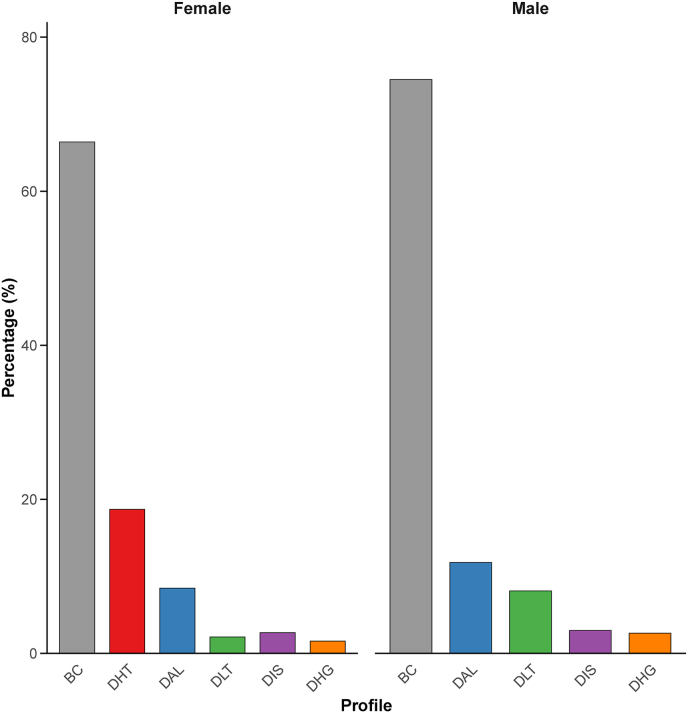
The proportions of each profile allocation. BC, baseline concordant; DAL, discordant adverse lipid; DHG, discordant hyperglycemic; DHT, discordant hypertensive; DIS, discordant inflammatory state; DLT, discordant liver transaminase.

The identified profiles included the baseline concordant (BC) profile showing cardiometabolic marker values that aligned with BMI-predicted ranges, discordant hypertensive (DHT) profile characterized by higher BP values than expected for their BMIs (only in females), discordant adverse lipid (DAL) profile characterized by higher TG and LDL cholesterol but lower HDL cholesterol, discordant liver transaminase (DLT) profile with higher ALT, discordant inflammatory state (DIS) profile with higher CRP, and discordant hyperglycemic (DHG) profile with higher blood glucose (Figure 1). The proportions of each profile allocation are shown in Figure 2. The BC profile represented the majority of both sexes, comprising 74.5% of males and 66.4% of females. The DHT profile was exclusively identified in females (18.7%). The DAL profile accounted for 11.8% of males compared with 8.46% of females. Less prevalent clusters included the DLT (8.12% male, 2.12% female), DIS (2.99% male, 2.70% female), and DHG (2.63% male, 1.59% female) profiles. The proportions of these profiles are similar to those observed in other European populations in Coral’s study.

### Association of the discordant profiles with risk of MASLD and LREs

**FIGURE 3:**
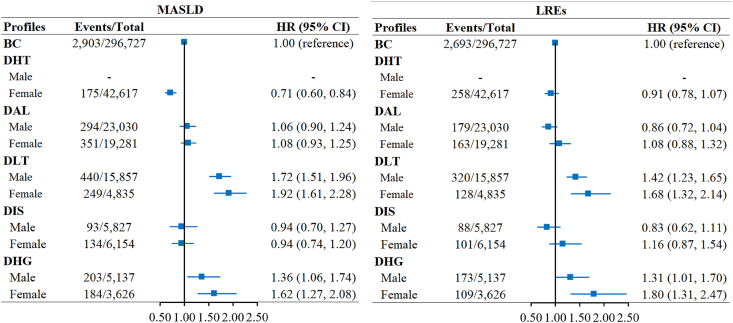
Sex-stratified associations of the discordant profiles with risk of incident MASLD and LREs. Cox models were adjusted for age, ethnicity, Townsend deprivation index, smoking, alcohol intake, healthy diet score, physical activity, sedentary time, use of antihypertensive agents, use of antidiabetic agents, use of lipid-lowering agents, cardiovascular disease, cancer, and cardiometabolic biomarkers. BC, baseline concordant; CI, confidence interval; DAL, discordant adverse lipid; DHG, discordant hyperglycemic; DHT, discordant hypertensive; DIS, discordant inflammatory state; DLT, discordant liver transaminase; HR, hazard ratio; LRE, liver-related event; MASLD, metabolic dysfunction–associated steatotic liver disease.

**FIGURE 4:**
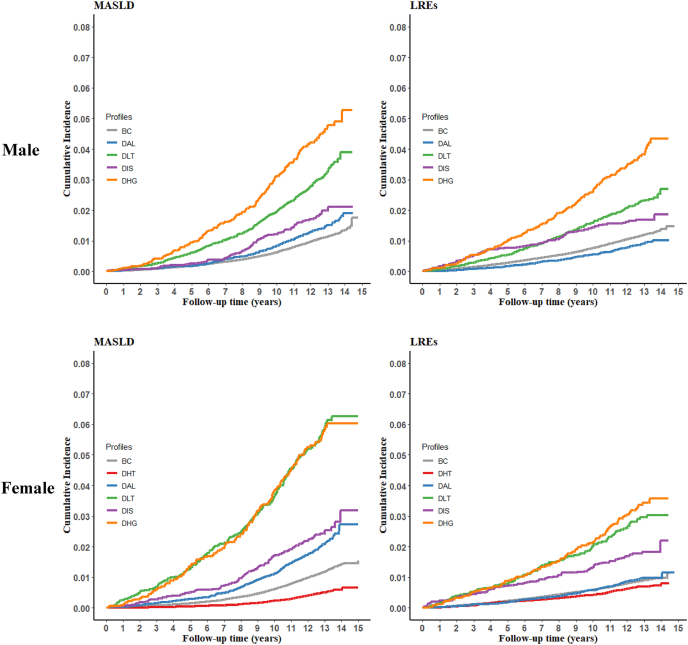
Cumulative incidence of MASLD and LREs across the different profiles. BC, baseline concordant; DAL, discordant adverse lipid; DHG, discordant hyperglycemic; DHT, discordant hypertensive; DIS, discordant inflammatory state; DLT, discordant liver transaminase; LRE, liver-related event; MASLD, metabolic dysfunction–associated steatotic liver disease.

During a median follow-up of 12 (interquartile range: 11–13) y, 2411 (1.24%) developed MASLD and 2249 (1.15%) developed LREs among 195,168 male participants; 2615 (1.15%) developed MASLD and 1963 (0.86%) developed LREs among 227,923 female participants. Figure 3 presents sex-stratified HRs (95% CIs) for MASLD and LREs across the identified profiles. Compared with the BC profile, the DHT profile exhibited a significant inverse association with MASLD (HR = 0.71; 95% CI: 0.60, 0.84) only in females, as the profile was not identified in males. In contrast, the DLT and DHG profiles had positive associations with risks of MASLD and LREs in both males and females. The multivariable HRs (95% CIs) of incident MASLD and LREs in the DLT profile were 1.72 (1.51, 1.96) and 1.42 (1.23, 1.65) in males and 1.92 (1.61, 2.28) and 1.68 (1.32, 2.14) in females. The corresponding HRs (95% CIs) in the DHG profile were 1.36 (1.06, 1.74) in males and 1.62 (1.27, 2.08) in females for MASLD and 1.31 (1.01, 1.70) in males and 1.80 (1.31, 2.47) in females for LREs. For the DAL and DIS profiles, no significant associations with the outcomes were observed. After controlling for false discovery rate, significant associations were not appreciably altered. Furthermore, the cumulative incidence of MASLD and LREs in both sexes was highest among participants in the DLT and DHG profiles (Figure 4).

### Predictive performance of the identified profiles

**FIGURE 5:**
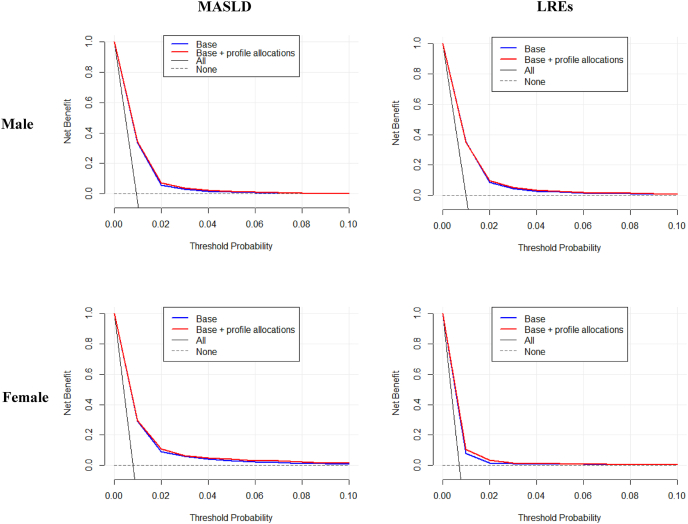
Decision curves of discordant profiles compared the prediction models with sociodemographic, lifestyle, clinical, and cardiometabolic biomarkers with models that additionally included profile estimations. LRE, liver-related event; MASLD, metabolic dysfunction–associated steatotic liver disease.

**FIGURE 6:**
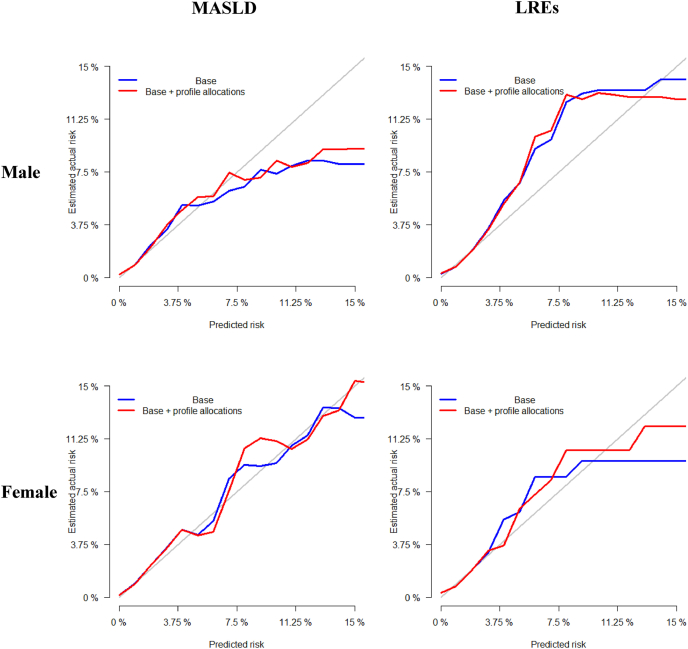
The calibration plots for predicting 10-y risks of incident MASLD and LREs in models with and without the different profiles. Base models included age, ethnicity, Townsend deprivation index, smoking, alcohol intake, healthy diet score, physical activity, sedentary time, use of antihypertensive agents, use of antidiabetic agents, use of lipid-lowering agents, cardiovascular disease, cancer, and cardiometabolic biomarkers. LRE, liver-related event; MASLD, metabolic dysfunction–associated steatotic liver disease.

The likelihood ratio test comparing models without and with profile allocations was all statistically significant (all *P* < 0.005). In males, the *c*-statistic, integrated discrimination improvement, and net reclassification improvement index were increased when adding the identified profiles to the reference model for MASLD and LREs. In contrast, the net reclassification improvement index for MASLD (−0.1178; 95% CI: −0.1561, −0.0896) and the *c*-statistic for LREs (difference: −0.0049; 95% CI: −0.0070, −0.0029) were decreased when adding the identified profiles to the reference model in females (Table 2). Figure 5 shows the decision curves of discordant profiles, and Figure 6 presents the calibration plots. In males, the DLT, DIS, and DHG profiles were retained in the Lasso models for both MASLD and LREs. In females, the DHT and DLT profiles were retained in the Lasso models for MASLD and LREs.

**TABLE 2:** Added predictive ability of the identified profiles predicting risks of MASLD and LREs

	Difference in *c*-statistic (95% CI)	*P* value	IDI (95% CI)	*P* value	NRI (95% CI)	*P* value
Male						
MASLD	0.0088 (0.0067, 0.0109)	<0.0001	0.0004 (0.0002, 0.0006)	<0.001	0.2149 (0.1782, 0.2516)	<0.0001
LREs	0.0044 (0.0030, 0.0059)	<0.0001	0.0003 (0.0002, 0.0005)	<0.001	0.1882 (0.1486, 0.2279)	<0.0001
Female						
MASLD	0.0080 (0.0061, 0.0100)	<0.0001	0.0008 (0.0005, 0.0010)	<0.0001	−0.1178 (−0.1561, −0.0896)	<0.0001
LREs	−0.0049 (−0.0070, −0.0029)	<0.0001	0.0001 (−0.0001, 0.0003)	0.42	0.0663 (0.023, 0.1097)	<0.01

Reference model included age, ethnicity, Townsend deprivation index, smoking, alcohol intake, healthy diet score, physical activity, sedentary time, use of antihypertensive agents, use of antidiabetic agents, use of lipid-lowering agents, cardiovascular disease, cancer, and cardiometabolic biomarkers.

Abbreviations: CI, confidence interval; IDI, integrated discrimination improvement; LRE, liver-related event; MASLD, metabolic dysfunction–associated steatotic liver disease; NRI, net reclassification improvement.

### Sensitivity analyses

The results were not appreciably altered when excluding participants diagnosed with MASLD or LREs within 2 y after recruitment (Supplementary Figure 2), estimating effects shifting 10% probability from the BC profile to each of the discordant profiles (Supplementary Figure 3), or accounting for the competing risk of death (Supplementary Figure 4).

## Discussion

In this large-scale population-based cohort study, we reproduced the 5 distinct profiles characterized by BMI-discordant cardiometabolic biomarkers originally identified by Coral et al. [9]. Moreover, we found that individuals with elevated liver transaminase (DLT profile) or hyperglycemia (DHG profile) disproportionate to their BMI exhibited higher risks of incident MASLD and LREs than those in other BMI-discordant profiles in both sexes. However, females with discordantly higher BP (DHT profile) demonstrated a lower risk of MASLD. In contrast, the DAL and DIS profiles did not show significant associations with MASLD and LREs. Furthermore, these profiles can add predictive value for risks of incident MASLD and LREs, possibly only in males.

Our results indicated that subpopulations with BMI-discordant cardiometabolic profiles had different risks of liver diseases. This finding may give early clues for identifying individuals with increased risks of MASLD and LREs. Moreover, our findings highlight the importance of considering heterogeneity in cardiometabolic profiles among individuals with similar BMIs when assessing risk of MASLD and LREs. Although obesity is a well-established risk factor for liver disease [1], the substantial variability in liver outcomes among individuals with comparable BMI levels suggests that other cardiometabolic factors play a critical role in disease progression. Our results are also supported by recent recommendations that solely BMI-based measures of obesity can both underestimate and overestimate adiposity and provide inadequate information about health at the individual level [[24], [25], [26]].

The DLT profile, characterized by disproportionately elevated ALT levels relative to BMI, was associated with a higher risk of both MASLD and LREs. This aligns with existing evidence that elevated ALT is a marker of hepatic injury or damage [27]. In addition, this profile has been cross-sectionally associated with a higher prevalence of liver failure [9]. Our finding is further corroborated by recent research indicating that MASLD with elevated ALT was positively associated with risk of severe liver diseases [28]. Similarly, the DHG profile, marked by higher-than-predicted blood glucose levels, was linked to an increased risk of MASLD and LREs. This finding is consistent with the well-documented role of insulin resistance and dysglycemia in the pathogenesis of MASLD [29]. Hyperglycemia exacerbates hepatic steatosis and fibrosis by promoting lipogenesis, oxidative stress, and inflammation [30]. Collectively, these findings suggest that individuals with elevated ALT or blood glucose relative to their BMI (even among those with BMI values traditionally considered healthful) may represent a high-risk subgroup that benefits from early screening and intensive lifestyle or pharmacological interventions to mitigate liver disease progression. This underscores the added value of integrating cardiometabolic biomarkers with BMI in risk stratification, in line with the recently proposed framework distinguishing clinical from preclinical obesity [24].

Interestingly, the female DHT profile (with BMI-discordantly elevated BP) was associated with a lower risk of MASLD. This observation is in line with previous findings that the DHT profile was associated with a lower risk of T2D compared with the BC profile [9]. Of note, the DHT profile also featured BMI-discordant HDL cholesterol elevation in addition to elevated BP, which may partly explain our results. Furthermore, it may reflect a unique metabolic phenotype in which hypertension may be driven by mechanisms less directly linked to hepatic steatosis, such as sodium retention rather than insulin resistance [31]. Estrogen may play an important modulatory role in such an association. Moreover, sex-specific trajectories in BP measures revealed that females exhibited a more pronounced rise in BP beginning as early as the third decade of life and continuing across the lifespan, independent of traditional metabolic risk factors [32]. This suggests that complex hormonal interplay may mitigate hepatic lipid accumulation despite androgenic activation. Further research is needed to elucidate the underlying mechanisms.

The lack of significant associations for the DAL and DIS profiles contrasts with Coral’s findings regarding T2D and CVD, except for the DAL profile, with a null association with CVD in males [9]. This suggests that dyslipidemia and systemic inflammation, when not accompanied by elevated liver enzymes or hyperglycemia, may be less predictive of liver-specific outcomes. Nevertheless, these profiles remain strongly linked to risks of T2D and CVD (in females) [9], underscoring the importance of phenotype-specific risk assessment for different diseases.

Although the UK Biobank is enriched for healthier females, adding the identified profiles to the reference model (including age, ethnicity, Townsend deprivation index, smoking, alcohol intake, healthy diet score, physical activity, sedentary time, use of antihypertensive agents, use of antidiabetic agents, use of lipid-lowering agents, CVD, cancer, BMI, WHR, SBP, DBP, blood glucose, SCR, ALT, CRP, HDL cholesterol, TG, and LDL cholesterol) can better outperform in predicting the future risk of MASLD and LREs in males than in females. This observed sex disparity may reflect intrinsic sex-specific metabolic heterogeneity, which may arise from differential hormonal regulation, body fat distribution patterns, or other sex-related pathophysiological factors that were not fully captured by the current study. Similarly, Coral et al. [9] also observed that the predictive accuracy of these clusters varies by sex. In particular, adding the DIS profile to CVD prediction models diminished accuracy in females but improved it in males [9]. Consistent with this, in our present study, the DIS profile was retained in the Lasso models for males not for females.

Our previous studies suggested that adherence to a healthy lifestyle (including not smoking, no alcohol intake, regular physical activity, short sedentary time, healthy diet, healthy sleep, and healthy weight) may lower risk of MASLD [33]. Thus, early lifestyle changes would be important for people who are subject to it once they have been deemed to have a high risk of liver diseases.

### Strengths and limitations

Our study has several strengths, including the large sample size, long follow-up, and comprehensive adjustment for potential confounders. The use of clustering techniques to capture BMI-discordant cardiometabolic profiles adds a novel dimension to risk stratification. More importantly, our findings suggest that precision prevention approaches can be effectively implemented without relying on costly high-throughput technologies, as individual stratification and targeted prevention can at times be done by juxtaposing more readily available clinical parameters to define “discordant” subtypes using machine learning methods.

However, some limitations should be acknowledged. First, MASLD was identified using ICD-10 codes, which may underestimate the true incidence. However, this likely prioritizes clinically relevant MASLD. Second, data on cardiometabolic biomarkers were collected once at baseline, so the effect of changes in these biomarkers and profiles over time on the outcomes could not be assessed. Third, the very low prevalence of some profiles, together with the low incidence of outcomes, may limit the precision of effect estimates. Fourth, as with any observational study, residual confounding cannot be ruled out, and causality cannot be inferred. Finally, the generalizability of our findings to other populations may be limited due to the UK Biobank cohort.

In conclusion, our study indicates that subclassifying BMI based on cardiometabolic biomarkers refines the prediction of MASLD and LREs. Individuals with disproportionately elevated liver enzymes or hyperglycemia relative to their BMI have higher risks than those with BMI-cordant cardiometabolic biomarkers. These findings support a precision medicine approach in liver disease prevention, moving beyond BMI alone to incorporate metabolic heterogeneity in risk assessment. Future studies should validate these profiles in diverse populations and explore targeted interventions for high-risk subgroups.

## Author contributions

The authors’ responsibilities were as follows – LQ, SZ: conceived and designed the research; SZ: analyzed the data and wrote the article; SZ, DEC, ZH, YB, TH, LQ: revised the manuscript critically for important intellectual content; and all authors: read and approved the final manuscript.

## Data availability

The data that support the findings of this study are available on application to the UK Biobank (application number 44430).

## Funding

This work was supported by grants from the National Natural Science Foundation of China (No. 82304128), the Young Elite Scientists Sponsorship Program by CAST (number 2023QNRC001), and the Fundamental Research Funds for the Central Universities (xzy012024141).

## Conflict of interest

The authors declare no conflict of interest.
