# Supplementary material for: Body Mass Index Subclassification and Future Risk of Metabolic Dysfunction–Associated Steatotic Liver Disease and Liver-Related Events
Source: J Nutr. 2026 Jan 29;156(4):101386. doi: 10.1016/j.tjnut.2026.101386 (PMC13084603; doi:10.1016/j.tjnut.2026.101386)
Supplement: Multimedia component 1 [file mmc1.docx]

**Online Supplementary Materials**

**Title: Body Mass Index Subclassification and Future Risk of Metabolic Dysfunction-Associated Steatotic Liver Disease and Liver-Related Events**

**First Author: Shunming Zhang**

| Page 2: | **Supplementary Figure 1.** Flowchart of study participants |
| --- | --- |
| Page 3: | **Supplementary Figure 2.** Sex-stratified associations of the discordant profiles with the risk of incident MASLD and LREs, excluding incident events that occurred within the first two years of follow-up |
| Page 4: | **Supplementary Figure 3.** Sex-stratified associations of the discordant profiles with the risk of incident MASLD and LREs, estimating effects shifting 10% probability from the baseline concordant profile to each of the discordant profiles |
| Page 5: | **Supplementary Figure 4.** Sex-stratified associations of the discordant profiles with the risk of incident MASLD and LREs, using Fine-Gray subdistribution hazard models that accounted for the competing risk of death |
| Page 6: | **Supplementary Table 1.** International Classification of Diseases, 10th revision codes for identifying liver-related events |
| Page 7: | **Supplementary Table 2.** The information on missing values for covariates |
| Pages 8-9: | **Supplementary Table 3.** Baseline characteristics of the study participants across validated profiles in males |
| Pages 10-11: | **Supplementary Table 4.** Baseline characteristics of the study participants across validated profiles in females |

**Supplementary Figure 1.** Flowchart of study participants. Abbreviations: LREs, liver-related events; MASLD, metabolic dysfunction-associated steatotic liver disease.


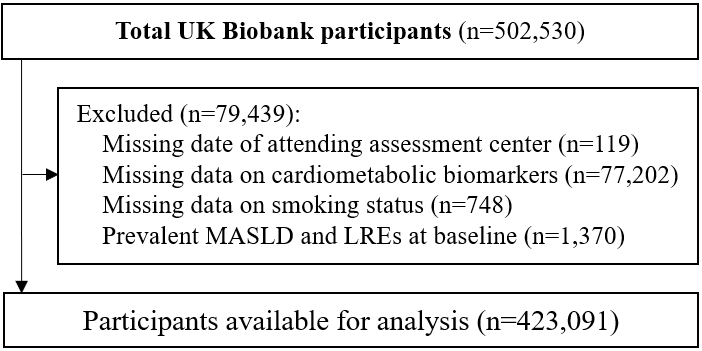


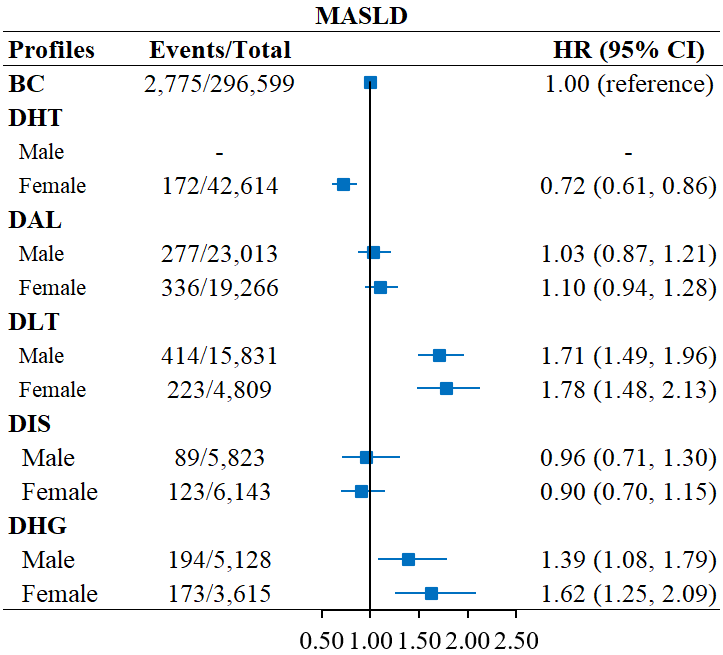

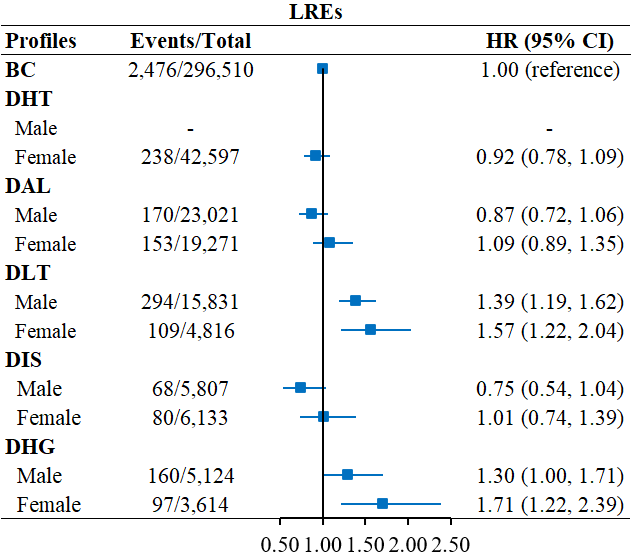
**Supplementary Figure 2.** Sex-stratified associations of the discordant profiles with the risk of incident MASLD and LREs, excluding incident events that occurred within the first two years of follow-up. Cox models were adjusted for age, ethnicity, Townsend deprivation index, smoking, alcohol intake, healthy diet score, physical activity, sedentary time, use of antihypertensive agents, use of antidiabetic agents, use of lipid-lowering agents, cardiovascular disease, cancer, and cardiometabolic biomarkers. Abbreviations: BC, baseline concordant; CI, confidence interval; DAL, discordant adverse lipid; DHG, discordant hyperglycemic; DHT, discordant hypertensive; DIS, discordant inflammatory state; DLT, discordant liver transaminase; HR, hazard ratio; LREs, liver-related events; MASLD, metabolic dysfunction-associated steatotic liver disease.

**Supplementary Figure 3.** Sex-stratified associations of the discordant profiles with the risk of incident MASLD and LREs, estimating effects shifting 10% probability from the baseline concordant profile to each of the discordant profiles. Cox models were adjusted for age, ethnicity, Townsend deprivation index, smoking, alcohol intake, healthy diet score, physical activity, sedentary time, use of antihypertensive agents, use of antidiabetic agents, use of lipid-lowering agents, cardiovascular disease, cancer, and cardiometabolic biomarkers. Abbreviations: CI, confidence interval; DAL, discordant adverse lipid; DHG, discordant hyperglycemic; DHT, discordant hypertensive; DIS, discordant inflammatory state; DLT, discordant liver transaminase; HR, hazard ratio; LREs, liver-related events; MASLD, metabolic dysfunction-associated steatotic liver disease.


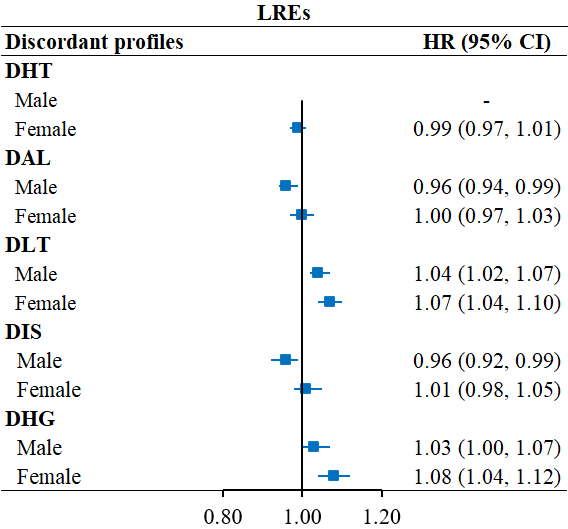

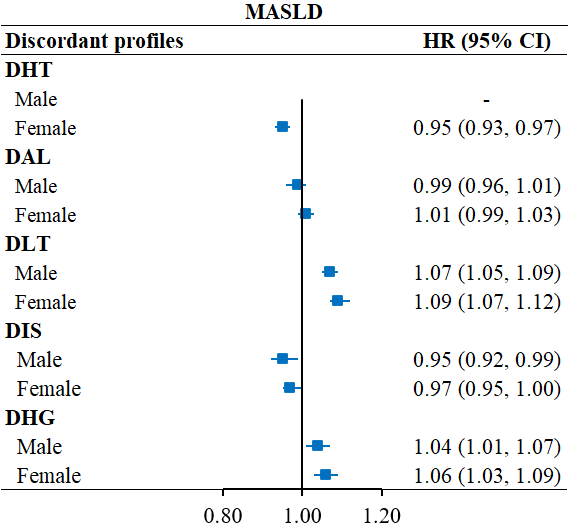


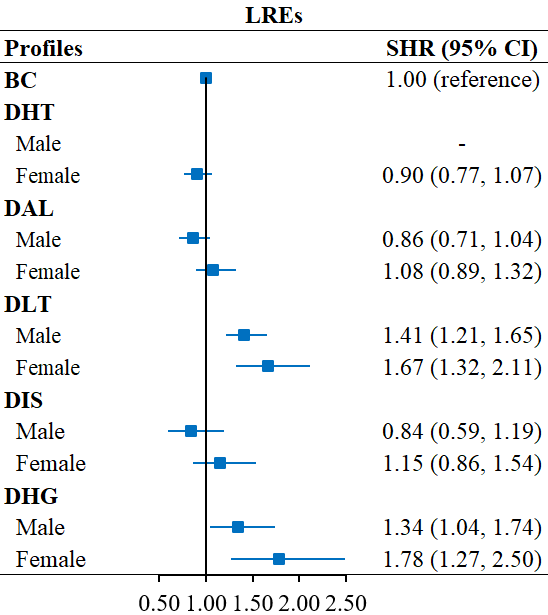

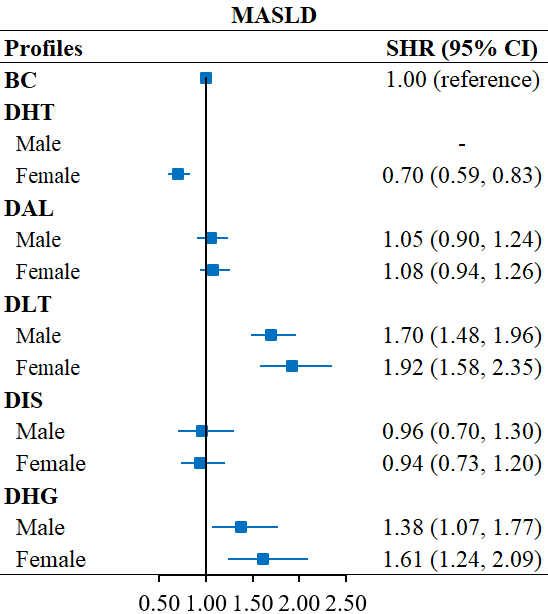
**Supplementary Figure 4.** Sex-stratified associations of the discordant profiles with the risk of incident MASLD and LREs, using Fine-Gray subdistribution hazard models that accounted for the competing risk of death. Models were adjusted for age, ethnicity, Townsend deprivation index, smoking, alcohol intake, healthy diet score, physical activity, sedentary time, use of antihypertensive agents, use of antidiabetic agents, use of lipid-lowering agents, cardiovascular disease, cancer, and cardiometabolic biomarkers. Abbreviations: BC, baseline concordant; CI, confidence interval; DAL, discordant adverse lipid; DHG, discordant hyperglycemic; DHT, discordant hypertensive; DIS, discordant inflammatory state; DLT, discordant liver transaminase; LREs, liver-related events; MASLD, metabolic dysfunction-associated steatotic liver disease; SHR, subdistribution hazard ratio.

| **Supplementary Table 1.** International Classification of Diseases, 10th revision codes for identifying liver-related events | |
| --- | --- |
| Diseases | Codes |
| Hepatocellular carcinoma | C22.0 |
| Hepatic decompensation | K74.6 |
| Ascites | R18 |
| Variceal hemorrhage | I98.3 |
| Hepatic encephalopathy | K72.9 |
| Hepatorenal syndrome | K76.7 |
| Liver transplantation | Z94.4 |
| Liver-related death (excluding MASLD) |  |
| Malignant neoplasm of the liver and intrahepatic bile ducts | C22.0, C22.7, C22.8 |
| Alcoholic liver disease | K70.1, K70.10, K70.11, K70.2, K70.30, K70.31, K70.4, K70.40, K70.41, K70.9 |
| Toxic liver disease | K71.7, K71.9 |
| Acute and subacute hepatic failure | K72.0, K72.1, K72.10, K72.11, K72.9, K72.90, K72.91 |
| Chronic hepatitis, not elsewhere classified | K73.0, K73.1, K73.2, K73.8, K73.9 |
| Hepatic cirrhosis or fibrosis | K74.6, I85.9, I98.2, I86.4, I85.0, I98.3, R18, K76.7, K76.6, K74.0, K74.1, K74.2 |
| Other inflammatory liver diseases | K75.8, K75.81, K75.89, K75.9 |
| Other liver diseases | K76.6, K76.7, K76.8, K76.81, K76.89, K76.9, R18.9, I85.9, I98.2, I98.3, I864A, K6581 |

| **Supplementary Table 2.** The information on missing values for covariates | | | | |
| --- | --- | --- | --- | --- |
| Covariates | Total | N | N miss | Percentage (%) |
| Ethnicity | 423,091 | 421,634 | 1,457 | 0.34 |
| Townsend deprivation index | 423,091 | 422,577 | 514 | 0.12 |
| Alcohol intake | 423,091 | 360,952 | 62,139 | 14.69 |
| Healthy diet score | 423,091 | 417,867 | 5,224 | 1.23 |
| Sedentary time | 423,091 | 422,596 | 495 | 0.12 |
| Physical activity | 423,091 | 342,409 | 80,682 | 19.07 |

| **Supplementary Table 3.** Baseline characteristics of the study participants across validated profiles in males ^1^ | | | | | |
| --- | --- | --- | --- | --- | --- |
| Characteristics | Profiles | | | | |
|  | BC | DAL | DLT | DIS | DHG |
| Number of participants | 145,317 | 23,030 | 15,857 | 5,827 | 5,137 |
| Age (years) | 56.94±8.23 | 55.82±8.02 | 55.18±7.97 | 57.94±8.20 | 58.72±7.41 |
| White ethnicity | 137238 (94.44) | 21801 (94.66) | 15001 (94.60) | 5526 (94.83) | 4559 (88.75) |
| Townsend deprivation index | -1.31±3.12 | -1.38±3.05 | -1.35±3.11 | -0.81±3.37 | -0.61±3.44 |
| Body mass index (kg/m^2^) | 27.65±4.29 | 27.86±3.29 | 28.48±3.76 | 28.19±4.96 | 30.49±5.25 |
| Waist circumference (cm) | 96.18±11.50 | 97.66±8.79 | 99.29±9.84 | 98.61±12.67 | 104.96±13.35 |
| Waist-to-hip ratio | 0.93±0.07 | 0.95±0.05 | 0.95±0.05 | 0.95±0.07 | 0.99±0.07 |
| Systolic blood pressure (mmHg) | 140.27±17.17 | 142.05±16.80 | 146.53±18.70 | 139.43±20.40 | 141.56±17.94 |
| Diastolic blood pressure (mmHg) | 83.35±9.52 | 85.92±9.78 | 90.05±11.40 | 83.16±11.88 | 81.42±10.53 |
| Blood glucose (mmol/L) | 5.03±0.77 | 4.89±0.59 | 5.01±0.64 | 5.02±0.74 | 11.43±3.61 |
| Serum creatinine (umol/L) | 82.37±20.45 | 80.49±10.70 | 79.32±9.76 | 81.06±14.57 | 76.93±15.52 |
| Alanine aminotransferase (U/L) | 24.21±9.31 | 28.35±10.59 | 54.88±28.79 | 23.13±11.13 | 33.58±18.21 |
| C-reactive protein (mg/L) | 1.94±2.19 | 1.63±1.22 | 1.83±1.57 | 20.18±12.60 | 2.58±2.97 |
| High-density lipoprotein cholesterol (mmol/L) | 1.33±0.31 | 1.05±0.17 | 1.26±0.28 | 1.18±0.29 | 1.08±0.27 |
| Low-density lipoprotein cholesterol (mmol/L) | 3.43±0.84 | 3.78±0.73 | 3.88±0.98 | 3.19±0.80 | 2.66±0.82 |
| Triglycerides (mmol/L) | 1.66±0.77 | 3.84±1.36 | 2.09±0.88 | 1.62±0.75 | 2.45±1.50 |
| Current smoking | 17998 (12.39) | 2876 (12.49) | 1779 (11.22) | 1022 (17.54) | 598 (11.64) |
| Alcohol intake (g/week) | 157.81±144.10 | 148.12±144.15 | 184.08±168.06 | 155.64±170.20 | 129.10±143.80 |
| Healthy diet score | 3.17±1.19 | 3.02±1.18 | 3.03±1.18 | 3.00±1.20 | 3.10±1.18 |
| Sedentary time (hours/day) | 5.21±2.60 | 5.48±2.66 | 5.59±2.75 | 5.53±2.83 | 6.06±3.07 |
| Physical activity |  |  |  |  |  |
| Low | 21239 (14.62) | 4169 (18.10) | 2989 (18.85) | 1189 (20.41) | 1264 (24.61) |
| Moderate | 60287 (41.49) | 9716 (42.19) | 6669 (42.06) | 2244 (38.51) | 1960 (38.15) |
| High | 41575 (28.61) | 5617 (24.39) | 3761 (23.72) | 1333 (22.88) | 991 (19.29) |
| Missing | 22216 (15.29) | 3528 (15.32) | 2438 (15.37) | 1061 (18.21) | 922 (17.95) |
| Cardiovascular disease | 14418 (9.92) | 1543 (6.70) | 1256 (7.92) | 877 (15.05) | 1226 (23.87) |
| Cancer | 9844 (6.77) | 1438 (6.24) | 919 (5.80) | 559 (9.59) | 350 (6.81) |
| Use of antihypertensive agents | 35351 (24.33) | 4408 (19.14) | 3442 (21.71) | 1730 (29.69) | 2934 (57.12) |
| Use of antidiabetic agents | 1284 (0.88) | 50 (0.22) | 58 (0.37) | 63 (1.08) | 1250 (24.33) |
| Use of lipid-lowering agents | 32842 (22.60) | 3830 (16.63) | 3053 (19.25) | 1412 (24.23) | 3530 (68.72) |
| Abbreviations: BC, baseline concordant; DAL, discordant adverse lipid; DHG, discordant hyperglycemic; DIS, discordant inflammatory state; DLT, discordant liver transaminase. | | | | | |
| ^1^ Data are expressed as mean ± standard deviation for continuous variables or as frequency (%) for categorical variables. | | | | | |

| **Supplementary Table 4.** Baseline characteristics of the study participants across validated profiles in females ^1^ | | | | | | |
| --- | --- | --- | --- | --- | --- | --- |
| Characteristics | Profiles | | | | | |
|  | BC | DHT | DAL | DLT | DIS | DHG |
| Number of participants | 151,410 | 42,617 | 19,281 | 4,835 | 6,154 | 3,626 |
| Age (years) | 56.27±8.16 | 56.35±7.66 | 56.84±7.56 | 55.96±7.19 | 57.14±8.06 | 58.62±7.46 |
| White ethnicity | 142941 (94.41) | 40764 (95.65) | 18154 (94.15) | 4630 (95.76) | 5775 (93.84) | 3194 (88.09) |
| Townsend deprivation index | -1.33±3.04 | -1.66±2.87 | -1.32±3.01 | -1.22±3.07 | -0.86±3.28 | -0.59±3.33 |
| Body mass index (kg/m^2^) | 27.09±5.25 | 25.89±4.34 | 27.46±4.01 | 28.78±5.08 | 29.67±6.89 | 31.18±6.88 |
| Waist circumference (cm) | 84.69±12.59 | 80.17±10.30 | 88.75±10.11 | 90.21±12.37 | 90.62±14.91 | 96.72±15.71 |
| Waist-to-hip ratio | 0.82±0.07 | 0.79±0.06 | 0.87±0.06 | 0.86±0.07 | 0.84±0.07 | 0.88±0.09 |
| Systolic blood pressure (mmHg) | 130.91±17.30 | 149.26±19.06 | 137.86±18.62 | 138.39±18.79 | 135.97±18.52 | 138.86±18.68 |
| Diastolic blood pressure (mmHg) | 78.23±8.98 | 88.36±9.06 | 82.69±9.88 | 84.23±10.33 | 81.68±10.37 | 79.61±10.55 |
| Blood glucose (mmol/L) | 4.97±0.70 | 4.94±0.43 | 4.99±0.58 | 5.08±0.79 | 5.05±0.73 | 10.77±3.44 |
| Serum creatinine (umol/L) | 65.04±13.52 | 62.57±8.18 | 62.13±9.26 | 62.52±9.71 | 63.83±11.72 | 66.39±34.05 |
| Alanine aminotransferase (U/L) | 19.16±8.04 | 16.97±4.98 | 21.52±8.29 | 71.20±37.77 | 19.02±8.47 | 26.12±14.63 |
| C-reactive protein (mg/L) | 2.31±2.57 | 1.40±1.28 | 2.29±1.88 | 3.53±4.01 | 20.97±11.24 | 4.45±5.45 |
| High-density lipoprotein cholesterol (mmol/L) | 1.55±0.32 | 1.93±0.37 | 1.27±0.21 | 1.50±0.39 | 1.45±0.36 | 1.34±0.38 |
| Low-density lipoprotein cholesterol (mmol/L) | 3.53±0.85 | 3.71±0.77 | 4.31±0.88 | 3.84±0.96 | 3.45±0.81 | 2.97±0.90 |
| Triglycerides (mmol/L) | 1.43±0.62 | 1.13±0.40 | 3.20±1.13 | 1.81±0.84 | 1.59±0.72 | 2.18±1.27 |
| Current smoking | 13290 (8.78) | 3583 (8.41) | 1931 (10.02) | 384 (7.94) | 729 (11.85) | 323 (8.91) |
| Alcohol intake (g/week) | 76.52±80.30 | 103.17±94.33 | 71.06±83.81 | 97.69±111.74 | 70.74±82.62 | 54.70±78.58 |
| Healthy diet score | 3.67±1.09 | 3.77±1.06 | 3.59±1.11 | 3.58±1.11 | 3.50±1.14 | 3.53±1.13 |
| Sedentary time (hours/day) | 4.39±2.17 | 4.15±2.00 | 4.59±2.20 | 4.71±2.28 | 4.75±2.34 | 4.88±2.51 |
| Physical activity |  |  |  |  |  |  |
| Low | 22014 (14.54) | 4959 (11.64) | 3133 (16.25) | 892 (18.45) | 1152 (18.72) | 748 (20.63) |
| Moderate | 61650 (40.72) | 17539 (41.15) | 7605 (39.44) | 1864 (38.55) | 2236 (36.33) | 1303 (35.93) |
| High | 34493 (22.78) | 11126 (26.11) | 4039 (20.95) | 942 (19.48) | 1111 (18.05) | 600 (16.55) |
| Missing | 33253 (21.96) | 8993 (21.10) | 4504 (23.36) | 1137 (23.52) | 1655 (26.89) | 975 (26.89) |
| Cardiovascular disease | 7437 (4.91) | 899 (2.11) | 742 (3.85) | 249 (5.15) | 379 (6.16) | 527 (14.53) |
| Cancer | 15145 (10.00) | 4169 (9.78) | 2141 (11.10) | 529 (10.94) | 772 (12.54) | 436 (12.02) |
| Use of antihypertensive agents | 25860 (17.08) | 6226 (14.61) | 3542 (18.37) | 1112 (23.00) | 1513 (24.59) | 1878 (51.79) |
| Use of antidiabetic agents | 730 (0.48) | 33 (0.08) | 41 (0.21) | 30 (0.62) | 39 (0.63) | 926 (25.54) |
| Use of lipid-lowering agents | 20565 (13.58) | 2482 (5.82) | 2248 (11.66) | 746 (15.43) | 750 (12.19) | 2189 (60.37) |
| Abbreviations: BC, baseline concordant; DAL, discordant adverse lipid; DHG, discordant hyperglycemic; DHT, discordant hypertensive; DIS, discordant inflammatory state; DLT, discordant liver transaminase. | | | | | | |
| ^1^ Data are expressed as mean ± standard deviation for continuous variables or as frequency (%) for categorical variables. | | | | | | |
